# Poster Session II A336 GASTROINTESTINAL MOTILITY IN CANNABIS USERS: A WIRELESS MOTILITY CAPSULE INVESTIGATION

**DOI:** 10.1093/jcag/gwaf042.336

**Published:** 2026-02-13

**Authors:** D Javidi, S Xue, E Tonzi, E Shah, M Curley

**Affiliations:** Medicine, Dartmouth Hitchcock Medical Center, Lebanon, NH; Medicine, Dartmouth Hitchcock Medical Center, Lebanon, NH; Medicine, Dartmouth Hitchcock Medical Center, Lebanon, NH; University of Michigan Michigan Medicine, Ann Arbor, MI; Medicine, Dartmouth Hitchcock Medical Center, Lebanon, NH

## Abstract

**Background:**

The cannabinoid receptors CB1 and CB2 are thought to play a role in gastrointestinal motility. Dronabinol, a non-selective cannabinoid agonist, has been shown to delay gastric emptying in humans(1). In mice, administration of a selective CB1 receptor agonist, was shown to delay gastric emptying and subsequent administration of a CB1 receptor antagonist reversed the effect(2).

**Aims:**

The goal of our study was to further explore the effects of cannabinoids on gastrointestinal motility in humans by conducting a retrospective analysis of a large cohort of patients who had undergone wireless motility capsule studies at our academic medical center.

**Methods:**

A retrospective analysis was conducted using data collected from the electronic medical record of patients who had undergone wireless motility capsule studies at Dartmouth-Hitchcock Medical Center from 2018-2022. Patients were divided into two cohorts of patients with active cannabis use and patients who did not use cannabis. Covariates of interest included age, sex, gastrointestinal symptoms, gastric emptying time (GET), small-bowel transit time (SBTT), colon transit time (CTT), and whole-gut transit time (WGTT), medications relevant to motility, and co-morbidities relevant to motility.

**Results:**

78 individuals with active cannabis use and 142 individuals with no history of cannabis use were included in the study. Individuals in the active cannabis use cohort were significantly younger **(p = 0.0001)** and more likely to complain of nausea **(p = 0.0001)** and vomiting **(p = 0.003)** than those without. Bloating was significantly more common in patients without active cannabis use **(p = 0.03)** but the prevalence of other gastrointestinal symptoms was similar between the two groups. The prevalence of usage of gastrointestinal motility affecting medications and comorbidities was not significantly different between the two groups. Individuals in the active cannabis use cohort had significantly longer gastric emptying times **(p = 0.02)** and a higher prevalence of delayed gastric emptying **(p = 0.006)** as compared to the group without cannabis use. No significant difference was appreciated with respect to small bowel or colon transit times between the two groups.

**Conclusions:**

Individuals with cannabis use display evidence of disordered gastrointestinal motility as measured by wireless motility capsule.

A336 Table 1: Gastrointestinal Transit Times Stratified by Cannabis Exposure

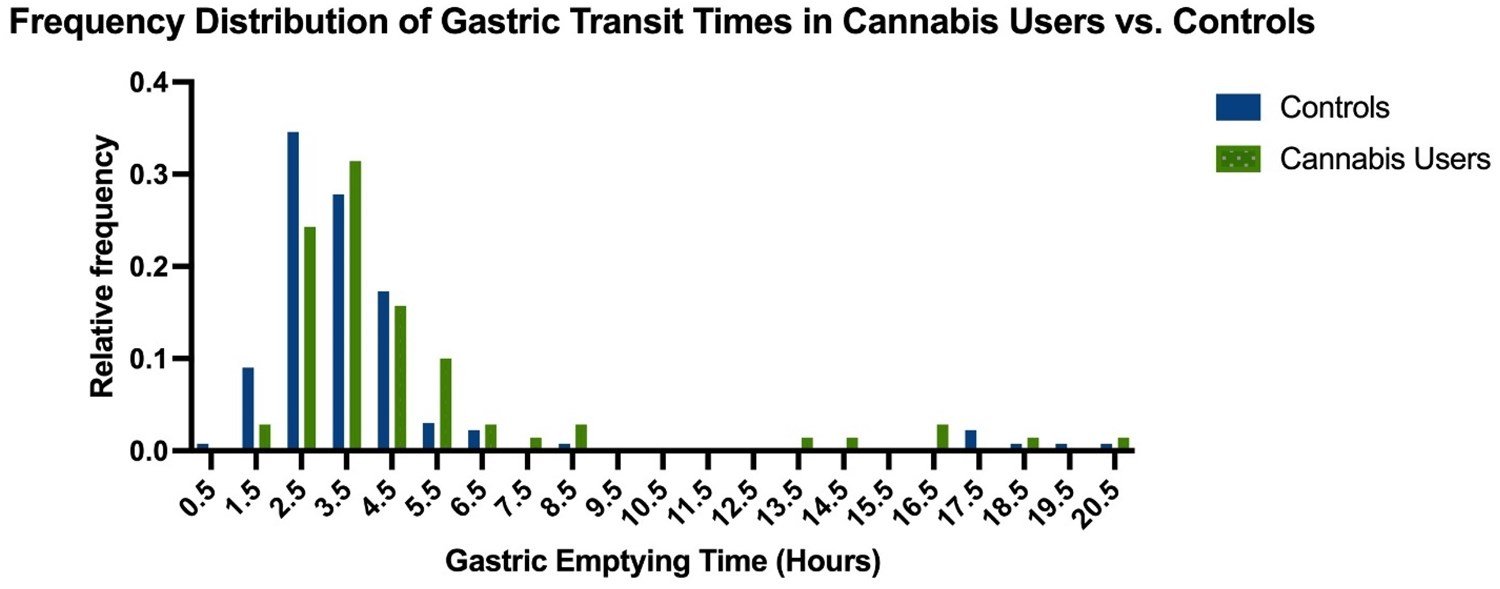

**Funding Agencies:**

None

**Table:** 

Transit Time	No Cannabis Use (n = 143)	Active Cannabis Use (n = 78)	p-value
Gastric Emptying Time	5.1 ± 0.7	7.9 ± 2.1	0.02
Small Bowel Transit Time	4.6 ± 0.2	4.2 ± 0.2	0.13
Colon Transit Time	47.3 ± 2.8	42.1 ± 3.8	0.17
Whole Gut Transit Time	52.1 ± 2.8	48.8 ± 4.1	0.29

